# Supplementary material for: Ocular movement abnormalities and ptosis after glaucoma surgery: A retrospective decade long analysis
Source: PLoS One. 2025 Oct 30;20(10):e0335074. doi: 10.1371/journal.pone.0335074 (PMC12574868; doi:10.1371/journal.pone.0335074)
Supplement: Attachment — Submitted filename: Response to reviewers.pdf [file pone.0335074.s003.pdf]

Dear Academic Editor and Reviewers os PlosOne,

we would like to thank the Academic Editor and all Reviewers for their valuable comments and suggestions, which have significantly contributed to improving the quality of our manuscript. We are are sorry for the delay in the submission of the revised manuscript.

We have carefully considered all observations and made the necessary changes. Below, we provide detailed responses to each point raised.

# Responses to the Academic Editor:

**Question 1**: Please present information on the study eye condition before glaucoma surgery in such old age patients, was ptosis present - even in mild degrees - before the glaucoma surgery? Or was it an entirely new development after glaucoma surgery?

**Response 1**: We have clarified this point on page 5, line 134, specifying that eyes considered eligible for the study had neither ptosis nor strabismus nor diplopia before surgery.

**Question 2**: Please mention if there were any elements of restrictive strabismus or ocular motility restriction generally.

**Response 2**: We have added this information on page 16, lines 283-284, as suggested.

**Question3**: Please present briefly details of the surgical procedures if available, mainly type of speculum used in the surgery, duration of surgery, level of training of surgeon, etc.

**Response 3**: We have integrated this information on page 6, lines 129-133, providing the requested details about the surgical procedures.

# Responses to Reviewer #1:

**Comment 1**: Abstract: Methods: 705 clinical records of patients; Results: 26 patients developed - Do not start with a number.

**Response 1** : We have corrected this formulation in the abstract, avoiding starting with a number.

**Comment 2**: The first two and half paragraphs in the introduction about the definition, prevalence and medical treatment of glaucoma represent unnecessary information.

**Response 2**: We believe that this information provides important context for understanding the relevance of our study, especially for those not versed in glaucoma subspecialities. However, we have revised these paragraphs to make them more concise and relevant as you suggested.

**Comment 3**: The patients were classified according to complications then according to the surgical procedure. The classification according to complications should be presented in the results, not in the methods section.

**Response 3**: As this is a retrospective observational study evaluating the prevalence of post-surgical complications, the classification was decided beforehand. Therefore, we consider it more appropriate to present it in the methods section, as it represents the main goal of the article.

**Comment 4**: Surgery without Conjunctival Opening - Ab Interno: Procedures such as Xen implant and cyclocryotherapy. Although cyclocryotherapy is done through a transconjunctival approach, it is an external procedure and could not be classified as an ab-interno one.

**Response 4**: This is correct; however, for the specific aim of this study, our primary classification criterion for surgeries was whether the conjunctiva was opened or not. Since both procedures (Xen implant and cyclocryotherapy) do not require conjunctival opening, we classified them in the same section. Thank you for your helpful suggestion - we have clarified this important distinction in the revised text.

**Comment 5**: There is no data regarding the use of superior rectus suture that may be used during different glaucoma surgeries as an alternative to corneal traction sutures to expose the upper part of the globe by some surgeons, and it is a very important cause of ptosis in these patients.

**Response 5**: We agree. We have added informations on the surgical approach used during glaucoma surgery, as suggested.

**Comment 6**: No data about the method of evaluation of ptosis, for example marginal reflex distance -1 or levator excursion evaluation.

**Response 6**: We have corrected this omission by adding details about the evaluation method of ptosis.

**Comment 7**: The statistical analysis methods should be mentioned in the methods section.

**Response 7**: We have moved this section as suggested.

**Comment 8**: The study included 705 medical records of patients who underwent filtering surgery. The study included 705 medical records of patients who underwent glaucoma surgery as cyclocryotherapy is not filtering surgery.

**Response 8**: We have applied the correction as suggested.

**Comment 9**: Table 1: characteristics by ptosis. The title of the table should be above not below it, to be applied for all tables.

**Response 9**: Thank you. We change the text in accordance with your suggestion

**Comment 10**: It is worth noting that 12 of these 19 strabismus cases (63.1%) occurred in patients who underwent bilateral filter surgery --> Filtering surgery.

**Response 10**: We have applied the correction as suggested.

# Responses to Reviewer #2:

**Comment 1**: From January 1, 2010, to December 31, 2020 is eleven not ten years.

**Response 1**: Thank you for pointing this out. We have corrected this error in the text and in the title of the manuscript.

**Comment 2**: In the abstract, it is expected to mention the percentage of patients who developed strabismus alone.

**Response 2**: We have added this information in the abstract on page 2, line 41, as suggested.

**Comment 3**: P31: "complications such as" has no place in the sentence.

**Response 3**: We have reformulated this sentence for greater clarity and coherence.

**Comment 4**: P100: "CLASS" deep sclerotomy needs to be explained before abbreviated.

**Response 4**: We have corrected according to the suggestion at line 101, providing the complete explanation before using the abbreviation.

**Comment 5**: P138-144: This paragraph would be more convenient if put under "Methods" not "Results".

**Response 5**: We have moved the paragraph to the Methods section as suggested.

**Comment 6**: Table 5 is better to be included in the results not the discussion.

**Response 6**: We have moved Table 5 to the Results section, as suggested.

**Comment 7**: The difference of the number of complicated groups in the tables from the stated number needs to be explained (31, 19 and 11 versus 26, 12 and 7 for ptosis, strabismus and both respectively).

**Response 7**: Thank you for this observation. We have adequately explained the discrepancies in the numbers in the text and tables.

**Comment 8**: P237-239: The numbers need to be mentioned and the percentage needs to be checked.

**Response 8**: We have verified and further explained the numbers and verified the percentages as you suggested.

**Comment 9**: P269-271: This paragraph needs to be supported by a reference.

**Response 9**: We have modified the text according to your suggestion, adding the appropriate references.

**Comment 10**: P279-287 and P293-295: these paragraphs are assumed to describe the work of the authors, citation is confusing.

**Response 10**: We have improved the placement of citations to avoid confusion.

**Comment 11**: The final conclusion is actually a part of the discussion but needs to be supported by references. Conclusion should be restricted to the results of outcome analysis.

**Response 11**: We have revised the conclusion of the manuscript as suggested, limiting it to the results of the analysis and adding the necessary references.

**Comment 12**: The results of all groups need to be described by the same sequence and by the same measures (numbers and percentages).

**Response 12**: The three groups are already described according to a fixed scheme in the results section:

1. First, the demographics of all groups are reported, with the total number of patients, then the sex distribution, mean age and whether there are statistically significant differences in age and sex distribution compared to the uncomplicated control group.
2. For each group, every surgery is analyzed: first describing how many patients underwent unilateral surgery and how many bilateral, then the average number of procedures per patient performed and whether there is a significant association in univariate and multivariate analysis with risk factors.
3. We then describe the results reported in Table 4 for the specific complication, citing the surgery combination most frequently associated with the complication.
4. Finally, we analyze the distribution of specific complication incidence according to the surgery, whether ab interno, ab externo, or with muscle manipulation.

# Responses to Reviewer #3:

We thank Reviewer #3 for the compliments on our informative study. No specific changes were proposed.

# Responses to Reviewer #4:

**Comment 1**: A good idea and work, some spelling mistakes must be corrected as in line 117 "Surgery with isolationa and".

**Response 1**: We have corrected the typographical error as suggested.

**Comment 2**: Line 130: "ocular alignment in the five primary gaze positions were documented from medical records." It was preferable to record the nine cardinal positions of gaze.

**Response 2**: We agree with your suggestion. The term "five" is a misprint. We recorded all nine cardinal positions in the chart, but we preferred not to include the amount of strabismus in the text because, in our opinion, it was not useful for the aim of this study.

**Comment 3**: The patient and method section devoid of assessment of ptosis (must be included).

**Response 3**: We have included the details on ptosis assessment as suggested.

**Comment 4**: Please provide more details on types of strabismus (angles of esotropia and exotropia), and association of oblique muscle dysfunction.

**Response 4**: These details were not described as they exceed the purpose of the manuscript, which is to describe the prevalence of complications after glaucoma surgery.

**Comment 5**: Please mention both visual acuity and amblyopia that may be contributing factors for development of strabismus.

**Response 5**: We agree with your suggestion and have modified the manuscript accordingly, including information on visual acuity and amblyopia as potential contributing factors to strabismus development.

**Comment 6**: Please mention the different degrees of ptosis in the affected patients.

**Response 6**: We have modified the text according to your suggestion, including the different degrees of ptosis observed.

We would like to thank all Reviewers once again for their valuable contributions, which have significantly improved the quality of our manuscript. We are confident that the changes made have adequately addressed all the issues raised.

Sincerely,

Prof. Aldo Vagge
