# Supplementary material for: Ocular movement abnormalities and ptosis after glaucoma surgery: A retrospective decade long analysis
Source: PLoS One. 2025 Oct 30;20(10):e0335074. doi: 10.1371/journal.pone.0335074 (PMC12574868; doi:10.1371/journal.pone.0335074)
Supplement: Attachment — Submitted filename: Response_to_Reviewers_auresp_2.pdf [file pone.0335074.s004.pdf]

Dear Editor and Reviewers,

We sincerely thank the reviewers for their continued engagement with our manuscript. We are pleased to see that Reviewer #3 considers all previous comments adequately addressed and finds the manuscript acceptable for publication.

Response to Reviewer #2

Comment 2: We respectfully acknowledge Reviewer #2's assessment regarding the technical soundness of our manuscript. While no specific technical concerns were detailed, we would like to address the scientific rigor and methodological validity of our study. This study represents the largest cohort study (n=705) examining ptosis and strabismus after glaucoma surgery published to date, with a 10-year retrospective analysis providing substantial follow-up data. Our comprehensive inclusion of diverse surgical techniques reflects real-world clinical practice, while clear inclusion and exclusion criteria minimize selection bias (lines 93-98 and 121-134).

Our data collection methodology is robust, employing standardized examination protocols by subspecialty experts, including both pediatric ophthalmologists experienced in strabismus evaluation and oculoplastic surgeons. We used objective measurements with established clinical parameters such as MRD1 and MRD2 for ptosis assessment and cover-uncover tests for strabismus evaluation. The systematic categorization of surgical procedures based on invasiveness and anatomical approach, combined with consistent follow-up protocols across the study period, ensures reliable data quality.

Comment: 3: The statistical analysis is appropriate and follows established methodological standards by an expert statistician with years of experience (Dr. Irene Schiavetti, University of Genoa). We employed univariate logistic regression to identify individual risk factors, followed by multivariate analysis to control for confounding variables. Effect sizes are reported with confidence intervals to provide clinical interpretability, and our sample size is adequate for detecting clinically meaningful differences. Our findings demonstrate statistically significant associations, including increased strabismus risk with higher number of surgeries (OR: 1.47, 95% CI: 1.00-2.14, p=0.048), significantly elevated risk with muscle isolation surgery (OR: 6.57, 95% CI: 1.71-21.65, p=0.003), association between younger age and strabismus development (OR: 1.14, 95% CI: 1.06-1.29, p=0.04), and distinct risk profile for combined complications (OR: 1.58, 95% CI: 1.01-2.25, p=0.02).

Our results demonstrate external validity through alignment with published literature. Our ptosis incidence rate (3.7%) is consistent with Park et al.'s reported range (1.64-12.50%), while our strabismus rate (1.7%) aligns with the Tube vs. Trabeculectomy Study findings (5% for drainage devices). The combined complications rate (1.0%) represents a novel finding given the limited prior data in this area. These findings provide significant clinical impact by identifying specific surgical techniques with higher complication rates, enabling evidence-based risk stratification for patient counseling, offering practical guidance for surgical decision-making, and contributing to our understanding of mechanism-specific complications.

We acknowledge the inherent limitations of retrospective design while emphasizing that this study design is appropriate and widely accepted for rare complication assessment, while prospective studies would be of interest, the low complication rates observed suggest that retrospective studies remain highly valuable, our large sample size over an extended timeframe provides robust evidence, and standardized examination protocols minimize detection bias. Our findings contribute meaningfully to the limited existing literature, as few large-scale studies examine these specific complications, most prior reports focus on single surgical techniques, our comprehensive approach across multiple procedures provides broader insights, and our results inform evidence-based surgical decision-making.

Response to Reviewer #3

We appreciate Reviewer #3's positive assessment that all previous comments have been adequately addressed and that our corrections were appropriate.

Comment 4: Our dataset is comprehensive, including complete demographic data across all patient groups, detailed surgical procedure documentation, systematic complications assessment, and long-term follow-up with a minimum of one year post-surgery. Outcomes are rigorously defined, with ptosis classified by severity using validated MRD measurements, strabismus confirmed by multiple examination techniques, diplopia symptoms systematically recorded, and bilateral complications appropriately categorized. We maintain statistical transparency through analyses performed using R statistical software, complete results tables (Tables 1-5), reported effect sizes and confidence intervals, and provision of our raw dataset to the journal for verification.

Minor Editorial Revisions

We will implement the specific editorial suggestions from Reviewer #2 in comment 6 in the new version of the manuscript:

Line 31: Revised objectives as suggested: "To evaluate the prevalence of ptosis, strabismus and the combination of both after glaucoma surgery and determine which kind of surgery is most likely to be linked to these complications."

Line 140: Added the missing text "and an oculoplastic surgeon."

Final conclusion (line 380): We understand that the stylistic choice suggested by Reviewer 2 is to limit the conclusion section to commenting on the numerical findings of the article. However, we deliberately chose to provide our conclusive analysis in the opening sentence of the conclusion paragraph, since the results and outcome analyses are extensively discussed in the individual sections, including statistical significance testing.
We opted to complement the final statement of the discussion with a series of clinically practical considerations, which we believe represent key points for the prevention and management of the surgical complications described in the article.

We acknowledge Reviewer 2's comment and we are grateful for the suggestion. We have therefore decided to report in the conclusion section the results of outcome analysis of only the current study with the incidence percentages of the complications observed in our cohort and move our considerations to the discussion.

We thank the reviewers for their constructive comments.

Conclusion

We sincerely thank the reviewers for their insightful suggestions that greatly contributed to enhancing the quality of our manuscript. We hope that this detailed explanation addresses any concerns regarding our research and demonstrates the robustness of our methodology as well as its clinical relevance. The study design is appropriate for the research question, the statistical analysis is rigorous and transparent, and our findings provide clinically relevant insights backed by robust evidence. Our work advances understanding of important but understudied complications in glaucoma surgery.

We hope this detailed explanation addresses any concerns about the technical soundness of our research and demonstrates the scientific merit of our contributions to the ophthalmologic literature.

Thank you for your consideration.

Sincerely,

Prof. Aldo Vagge

Dr. Carlo Catti
